# Identification of the fructose transporter GLUT5 (*SLC2A5*) as a novel target of nuclear receptor LXR

**DOI:** 10.1038/s41598-019-45803-x

**Published:** 2019-06-26

**Authors:** Irene Zwarts, Tim van Zutphen, Janine K. Kruit, Weilin Liu, Maaike H. Oosterveer, Henkjan J. Verkade, N. Henriette Uhlenhaut, Johan W. Jonker

**Affiliations:** 1 Section of Molecular Metabolism and Nutrition, Department of Pediatrics, University of Groningen, University Medical Center Groningen, Hanzeplein 1, 9713 GZ Groningen, The Netherlands; 2 Molecular Endocrinology, Institute for Diabetes and Cancer IDC, Helmholtz Zentrum München, Ingolstädter Landstr. 1, Neuherberg, 85764 Germany; 3 Gene Center, Ludwig-Maximilians-Universität München (LMU), Feodor-Lynen-Straße 25, Munich, 81377 Germany

**Keywords:** Molecular biology, Endocrinology

## Abstract

Fructose has become a major constituent of our modern diet and is implicated as an underlying cause in the development of metabolic diseases. The fructose transporter GLUT5 (SLC2A5) is required for intestinal fructose absorption. GLUT5 expression is induced in the intestine and skeletal muscle of type 2 diabetes (T2D) patients and in certain cancers that are dependent on fructose metabolism, indicating that modulation of GLUT5 levels could have potential in the treatment of these diseases. Using an unbiased screen for transcriptional control of the human *GLUT5* promoter we identified a strong and specific regulation by liver X receptor α (LXRα, NR1H3). Using promoter truncations and site-directed mutagenesis we identified a functional LXR response element (LXRE) in the human *GLUT5* promoter, located at −385 bp relative to the transcriptional start site (TSS). Finally, mice treated with LXR agonist T0901317 showed an increase in Glut5 mRNA and protein levels in duodenum and adipose tissue, underscoring the *in vivo* relevance of its regulation by LXR. Together, our findings show that LXRα regulates GLUT5 in mice and humans. As a ligand-activated transcription factor, LXRα might provide novel pharmacologic strategies for the selective modulation of GLUT5 activity in the treatment of metabolic disease as well as cancer.

## Introduction

The increasing prevalence of obesity is cause for public health concern since it is a major risk factor for the development of chronic metabolic diseases including type 2 diabetes (T2D), cardiovascular disease and certain types of cancer. The obesity pandemic is primarily attributed to our modern lifestyle of excessive calorie intake and relative inactivity. Fructose may be an important contributor to this pandemic as excessive consumption of fructose but not glucose has been shown to increase visceral adiposity and insulin resistance in overweight/obese humans^1^. In addition, fructose consumption has dramatically increased in the past 50 years to more than 10% of our daily calorie intake, mainly in the form of sucrose and inexpensive corn-based sweeteners such as high-fructose corn syrup (HFCS) that were introduced in the early 1970’s^2–4^.

Because of differences in absorption and metabolism between fructose and glucose, they have unique effects on the development of specific features of the metabolic syndrome. For example, whereas glucose is used as a fuel by all cells in the body and its plasma levels are tightly controlled by insulin, fructose is mainly taken up and metabolized by the intestine and liver, independently of insulin. In addition, since fructose doesn’t stimulate secretion of insulin, leptin or other satiety hormones, it is a much weaker suppressor of appetite than glucose^1,5–7^. In the liver, glucose is metabolized via phosphofructokinase (PFK), a rate-limiting step in the glycolytic pathway and subject to strong feedback inhibition. Fructose, on the other hand, can bypass this PFK regulatory block via fructokinase or ketohexokinase (KHK), allowing continued glycolytic flux independent of cellular energy status. This uncontrolled hepatic fructose metabolism increases lipogenesis and, consequently the development of non-alcoholic fatty liver disease (NAFLD)^7,8^. Another consequence of this lack in feedback regulation of fructolysis is a rapid depletion of ATP, resulting in increased uric acid production (hyperuricemia). Hyperuricemia in turn is known to cause hypertension and gout but is also an independent risk factor for metabolic syndrome, cardiovascular disease and the development of chronic kidney disease (CKD)^9^.

Besides the deleterious metabolic effects of fructose, its intestinal malabsorption, also known as dietary fructose intolerance, is a common problem, particularly in babies and patients with gastrointestinal disorders. Incomplete fructose absorption from the small intestine causes bloating, abdominal pain and diarrhea due to bacterial fructose fermentation in the colon^10,11^. Acute feeding studies have shown that almost one third of patients with irritable bowel syndrome (IBS) suffer from fructose malabsorption and benefit from a fructose-restricted diet^12,13^.

Fructose transport is mediated by members of the GLUT-family of facilitated sugar transporters encoded by the solute carrier family 2A (*SLC2A)* genes, which are essential for intestinal fructose absorption and fructose metabolism (Reviewed in^14^). GLUT5 (SLC2A5) is a high-affinity fructose transporter primarily expressed at the apical surface of intestinal epithelial cells but also at lower levels in testis, kidney, brain, skeletal muscle and white adipose tissue (WAT)^15^. GLUT5 is the only GLUT-member that has exclusive affinity for fructose and is essential for first-pass intestinal fructose absorption and generation of fructose-induced hypertension based on studies using Glut5 deficient mice^16^. The main inducers of *GLUT5* expression are fructose and to a lesser extent glucose, yet the underlying molecular mechanism of regulation by these sugars is largely unknown. Fructose transport from the intestine to the blood and uptake by the liver is mediated by GLUT2 (SLC2A2), a high-capacity, glucose-dependent fructose co-transporter primarily localized on the basolateral membrane of enterocytes and hepatocytes.

Under normal conditions, intestinal GLUT5 expression is low in neonates, explaining their sensitivity to fructose malabsorption, but in experiments with rats it has been shown that its expression can be induced by thyroid hormone (triiodothyronine, T_3_) and glucocorticoids through activation of the thyroid hormone receptor (THR) and the glucocorticoid receptor (GR), respectively, both members of the nuclear receptor (NR) family of ligand-activated transcription factors^17,18^. Whereas *GLUT5* expression was confirmed to be regulated by THR activation *in vitro*, T_3_ treatment *in vivo* only increased intestinal *Glut5* mRNA in rat pups younger than 22 days, suggesting that T_3_ has a role in intestinal maturation^18–20^. The intestine of neonatal rats was also sensitized to fructose-induced GLUT5 expression by the corticosteroid and GR ligand dexamethasone^17,21^.

Under pathophysiological conditions, GLUT5 expression was shown to be upregulated in the intestine and in skeletal muscle of T2D patients, and this could be reversed in skeletal muscle by treatment with pioglitazone, an insulin sensitizing drug of the thiazolidinedione (TZD) class of anti-diabetic drugs^22,23^. Moreover, GLUT5 expression is also overexpressed in certain cancers that are highly dependent on fructose uptake and metabolism such as breast cancer^24,25^, acute myeloid leukemia^26^ and pancreatic cancer metastasis^27^, providing cancer cells a route to increase the energy delivery in order to match increased energy demand caused by uncontrolled proliferation^28,29^. Despite recent focus on the impact of fructose on metabolic homeostasis and cancer, the regulatory mechanisms involved are still poorly understood.

In the current study, we identified the sterol-responsive NR liver x receptor α (LXRα) as a novel transcriptional regulator of both human and mouse GLUT5. Here we characterized the response element in the human *GLUT5* promoter, responsible for the interaction with LXRα and investigated in mice treated with a specific agonist how LXR-activation affects GLUT5 expression *in vivo*.

## Results

### Promoter regulation of the human fructose transporter GLUT5 by LXRα

**Figure 1:**
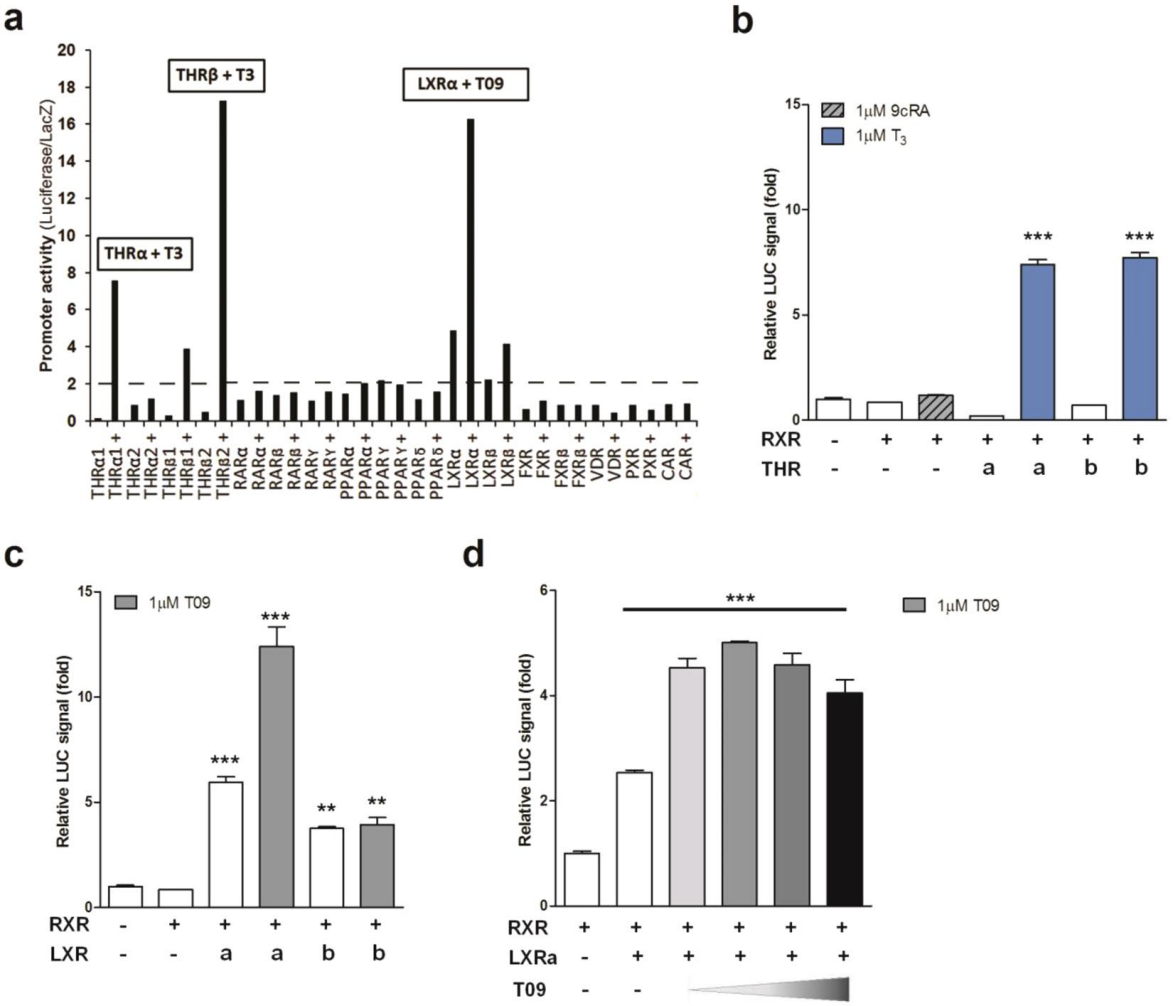
Transcriptional regulation of the human *GLUT5* promoter by nuclear receptors LXR and THR. Luciferase reporter analysis of the *hGLUT5* promoter for regulation by (**a)** Nuclear Receptors heterodimerizing with RXR; (**b**) THRα, THRβ; (**c)** LXRα and LXRβ. CV-1 cells were co-transfected with the proximal human *GLUT5* promoter (−900/+3 bp) and expression plasmids for the different NRs in combination with equal amounts of heterodimeric partner RXRα. Y-axis indicates luciferase activity relative to LacZ and normalized to the basal *hGLUT5* promoter activity. Ligand-dependent activation of THR and LXR was tested by addition of T_3_ (1 μM) or T09 (1 μM), respectively, at 24 h after transfection. (**d)** Dose-dependent stimulation of LXRα-mediated *hGLUT5* promoter regulation by LXR ligand T0901317.

Using a luciferase reporter screen for the transcriptional regulation by ligand-activated members of the NR-family that heterodimerize with RXR, we observed a strong and specific regulation of the human *GLUT5* promoter (−900/+3 bp relative to the transcription start site) by thyroid hormone receptors THRα and -β and LXRα in CV-1 cells (Fig. 1a). The regulation of the human *GLUT5* promoter by THRα and -β has previously been described^19^. RXRα, the heterodimeric partner of THR, alone did not result in a significant increase in luciferase signal, neither in the presence or absence of its ligand 9-cis-Retinoic acid (9cRA, 1 µM) (Fig. 1b). THRs are known to act as transcriptional repressors in the absence of their ligand, thyroid hormone (T_3_). Indeed, co-transfection of THRα together with RXRα resulted in an approximately 80% repression of the *GLUT5* promoter activity, in the absence of T_3_ (Fig. 1b)^30^. In the presence of T_3_ (1 μM), THRs act as transcriptional activators and in line with this we observed an about 7-fold increased *GLUT5* promoter activity by either THRα or -β as compared to basal promoter activity (Fig. 1b).

The regulation of *GLUT5* by LXR has not been reported previously. Co-transfection of LXRα together with its heterodimeric partner RXRα resulted in an activation of about 6-fold, which could be further stimulated to about 12-fold by addition of the synthetic LXR ligand T0901317 (T09) with an optimal concentration of 1 µM (Fig. 1c,d). In contrast, LXRβ/RXRα was less potent and resulted only in an about 3-fold activation of the GLUT5 promoter, and this could not be further increased by the addition of T09 (Fig. 1c). The regulation of *GLUT5* by LXRα was not specific to CV-1 cells as we also observed this in HEK293AD cells (Fig. S2). Together, these results demonstrate that LXRα is a strong and specific transcriptional regulator of the human *GLUT5* gene.

### Identification of functional LXR-responsive DR4 element in the human GLUT5 promoter

**Figure 2:**
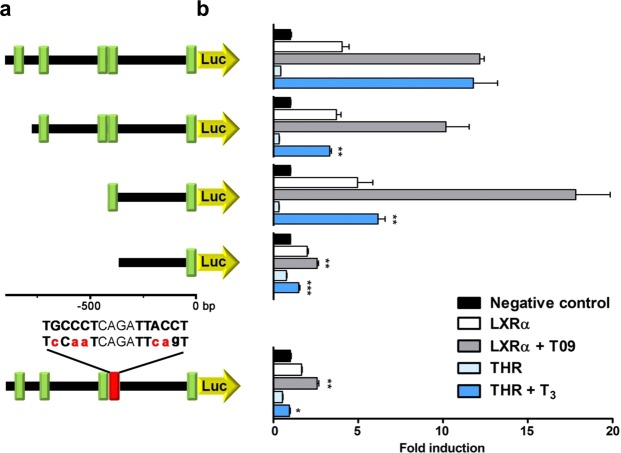
Identification of the functional LXR-responsive element in the human *GLUT5* promoter. **(a)** Overview of the promoter truncations used to localize the functional LXRE in the proximal human *GLUT5* promoter (−900/+3 bp). Predicted LXREs are represented by green boxes. The red box represents a site-directed point mutation in the functional LXRE. (**b**) Luciferase reporter analysis of the *hGLUT5* promoter truncations and mutant for regulation by LXR and THR. CV-1 cells were co-transfected with the indicated *hGLUT5-luc* constructs and expression plasmids for LXRα or THRα in combination with equal amounts of heterodimeric partner RXRα. X-axis indicates luciferase activity relative to LacZ and normalized to the negative control (NC = basal *hGLUT5* promoter activity). Ligand-dependent activation of LXR and THR was tested by addition of T09 (1 μM) or T_3_ (1 μM), respectively, at 24 h after transfection. For statistics, different truncations/mutations were compared for each receptor in the presence of ligand.

Next, we investigated the mechanism by which LXRα regulates the human *GLUT5* promoter. By *in silico* promoter analysis using MatInspector (Genomatix) to predict putative transcription factor binding sites, we identified 4 putative LXR response elements (LXREs) consisting of direct repeats of the consensus hexanucleotide sequence AGGTCA with a spacing of 4 (DR4) in the 900 bp human *GLUT5* promoter. To localize the essential DR4 element for the regulation by LXR, we generated three promoter truncations spanning −783 bp, −429 bp and −374 bp to +3 bp, respectively of the *hGLUT5* promoter sequence and cloned these in the pGL4-luc reporter plasmid (Fig. 2a). Whereas responsiveness to LXR activation was retained in the −783/+3 and −429/+3 truncations, this was almost completely abolished in the −374/+3 truncation. Based on these results, we conclude that the DR4 element essential for the regulation by LXRα is located within the promoter region between −429 and −374 bp (Fig. 2b). To further investigate the binding of LXR to this element we mutated both hexanucleotide repeats of this DR4 using site-directed mutagenesis, resulting in a change of sequence from the wild-type 5′-**TGCCCT**CAGA**TTACCT**-3′ to mutant 5′-**TcCaaT**CAGA**TTcagT**-3′. Similar to the −374/+3 truncation, activation by LXR was completely abolished in this mutant, indicating that this DR4 located on −385 bp indeed functions as a *bona fide* LXRE (Fig. 2b). Interestingly, this binding site has previously been described as a Thyroid hormone receptor response element (TRE)^19^. Indeed, in the −374/+3 promoter truncation and the mutated DR4 *GLUT5* promoter, also the THR regulation was abolished (Fig. 2b). To further investigate this regulation, we performed a competitive reporter assay and showed that LXRα and THRβ interfere with each other’s ability to activate the reporter, suggesting that both nuclear receptors compete for binding (Fig. S3).

### Regulation of the mouse *Glut5* promoter by LXRα

Sequence alignment of the human, mouse and rat proximal *GLUT5* promoters (500 bp upstream of the ATG start codon) showed a 66% similarity between human and rodent (mouse or rat), and a 87% similarity between mouse and rat *Glut5* promoters (Fig. S4a). To determine whether the regulation of the human *GLUT5* promoter by LXR is conserved in mice, we cloned the mouse *Glut5* promoter into the pGL4-luc reporter plasmid. Since the mouse *Glut5* gene is alternatively transcribed in somatic and germ cells, we cloned the sequence −1014 bp to +3 bp relative to the ATG start codon, as described for somatic cells^31^.

**Figure 3:**
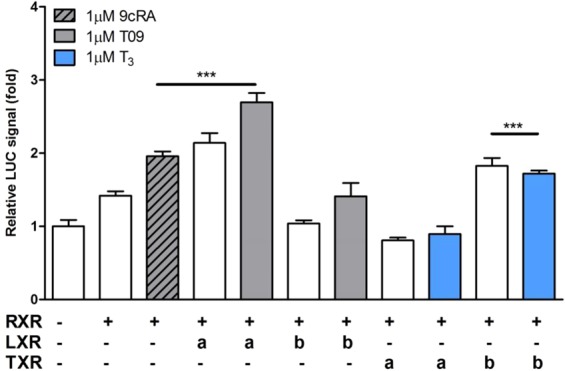
Transcriptional regulation of the mouse *Glut5* promoter by nuclear receptors LXR and THR. Luciferase reporter analysis of the *mGlut5* promoter for regulation by nuclear receptors LXRα, LXRβ, THRα and THRβ. CV-1 cells were co-transfected with the proximal mouse *Glut5* promoter (−1014/+3 bp) and expression plasmids for the different NRs in combination with equal amounts of heterodimeric partner RXRα. Y-axis indicates luciferase activity relative to LacZ and normalized to the basal *hGLUT5* promoter activity. Ligand-dependent activation of LXR and THR was tested by addition of T09 (1 μM) or T_3_ (1 μM), respectively, at 24 h after transfection.

Co-transfection of the mouse *Glut5* reporter construct with LXRα/RXRα expression plasmids resulted in an approximately 3-fold induction of luciferase activity and this was only slightly further stimulated by T09, both in CV-1 cells (Fig. 3) and in HEK293AD cells (Fig. S2). This induction was isoform-specific, as it was not observed for LXRβ. In addition, treatment with 1 μM 9cRA in the presence of RXRα resulted in an about 2-fold induction of the promoter activity, highlighting the role of RXR activation in mouse *Glut5* transcriptional regulation. Unlike *hGLUT5*, *mGlut5* did not appear to be substantially regulated by THRα or –β, either in the presence or absence of T_3_, although a small (<2-fold) increase was observed for THRβ (Fig. 3). The genomic binding of LXRα to the GLUT5 promoter was supported by publicly available chromatin immunoprecipitation sequencing (ChIP-seq) data in primary mouse macrophages (Gene Transcription Regulation Database, http://gtrd.biouml.org), in which an LXR binding site was identified at 1765 bp upstream of the *Glut5* transcription start site (TSS) for germ cells. A second LXR binding site was identified at 155 bp upstream of the TSS for somatic cells (Fig. S4b). Together, these results show that the regulation of the *GLUT5* promoter by LXR is conserved between humans and rodents and indicate that the mouse can be used as a model to study this process.

### *In vivo* regulation of Glut5 expression by LXR in mice

**Figure 4:**
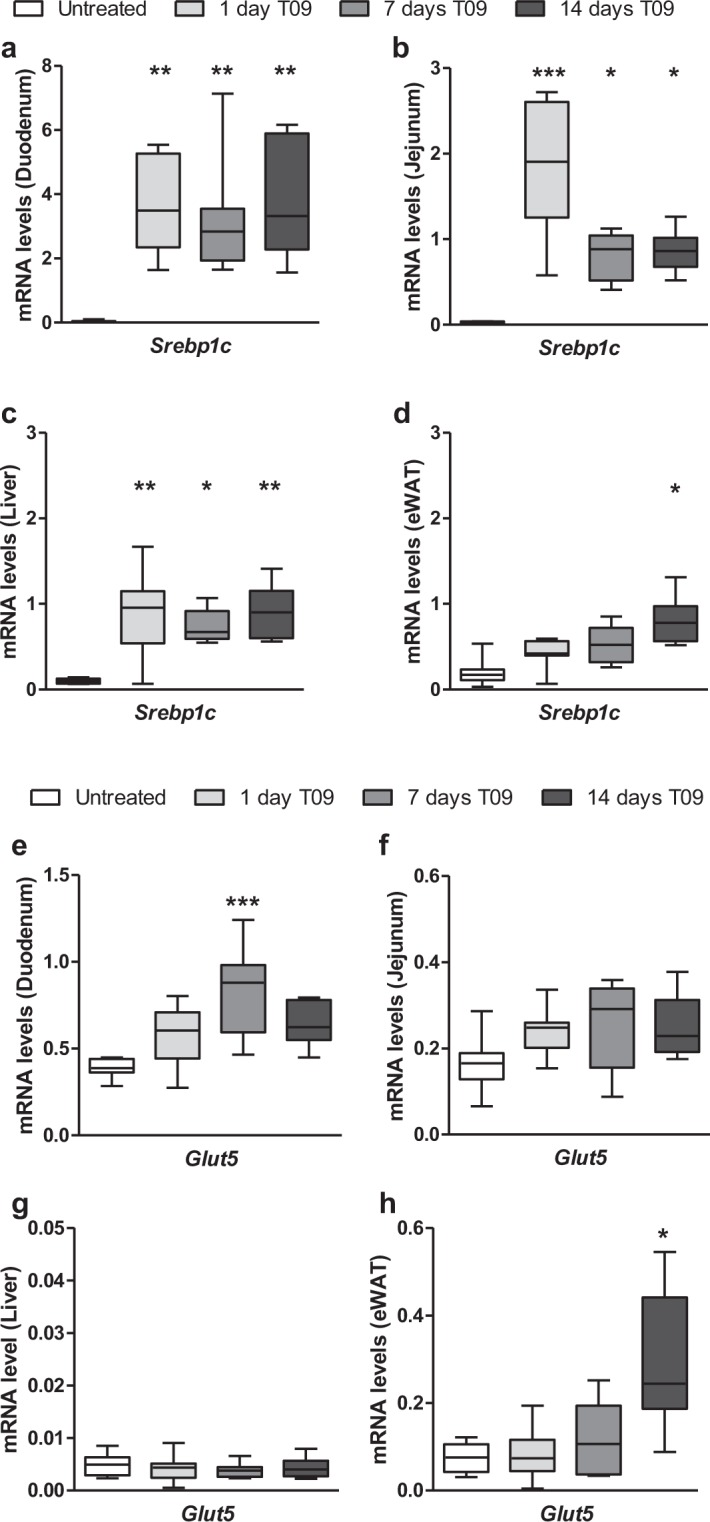
Effect of LXR-activation by T0901317 treatment on *Glut5 and Srebp1c* expression in mice. mRNA levels of *Srebp1c* and *Glut5* in (**a**,**e)** duodenum, (**b,f)** jejunum, (**c,g**) liver and (**d,h**) eWAT in wild-type mice treated for 1, 7 or 14 days with 0.015% T09 supplemented in their standard chow diet (n = 5–8, y-axis; normalized to *36B4* universal reference gene). Data are presented as boxplots (middle line, median; box, 25th–75th percentiles; whiskers, 5th–95th percentiles).

To investigate a potential role for LXR-mediated regulation *in vivo*, we determined the mRNA and protein levels of *Glut5* in tissues of male C57BL/6J mice that were treated with T09 (30 mg/kg/d) supplemented in standard chow diet for 1, 7 or 14 days. As expected T09 increased hepatic triglyceride levels but did not have a significant effect on food intake and total body weight (Fig. S5a–c). As a positive control for the effect of T09 treatment on LXR-mediated transcription we determined mRNA levels of sterol regulatory element-binding protein 1c (Srebp1c), a well-described LXR target gene^32,33^. *Srebp1c* mRNA levels were strongly increased upon T09 treatment in the small intestine (duodenum and jejunum) and liver after 1, 7 and 14 days of T09 treatment (Fig. 4a–d). In epididymal white adipose tissue (eWAT), there was also an increase in *Srebp1c* mRNA by T09 treatment, but this only reached significance after 14 days (Fig. 4h).

**Figure 5:**
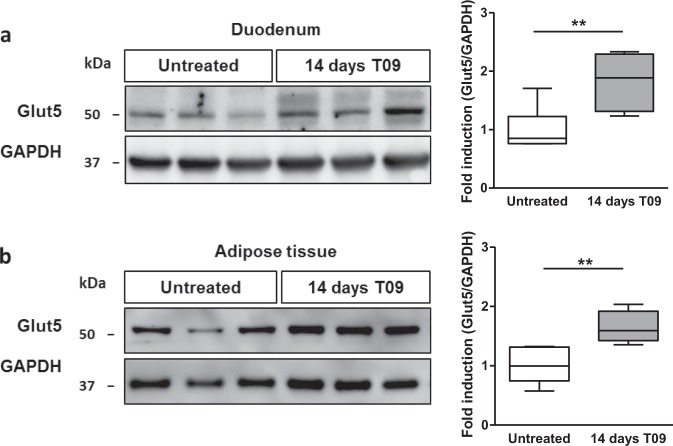
Effect of LXR-activation by T09 treatment on Glut5 protein levels in mice. Representative western blot analysis and quantification of Glut5 and Gapdh protein levels in (**a)** duodenum and (**b)** eWAT in wild-type mice treated for 14 days with 0.015% T09 supplemented in their standard chow diet (n = 4–6). Western blot quantification is depicted as boxplots (middle line, median; box, 25th–75th percentiles; whiskers, 5th–95th percentiles).

*Glut5* expression was highest in the duodenum and both mRNA and protein levels were significantly increased after 7 days of treatment with T09 (Figs 4e, 5a). In jejunum *Glut5* mRNA only showed a trend towards an increase but this was not statistically significant (Fig. 4f). *Glut5* expression levels were low and undetectable in liver and skeletal muscle, respectively, and did not change upon T09 treatment (Fig. 4g, not shown). Interestingly, *Glut5* expression both, at the mRNA and protein level, was also increased in eWAT of mice treated with T09 for 14 days, showing a similar pattern as observed for *Srebp1c* (Figs 4h, 5b).

## Discussion

Despite recent efforts in fructose research, many regulatory mechanisms involved in fructose absorption and metabolism, as well as the impact of dietary fructose in health and disease, have remained unclear. GLUT5 is indispensable for intestinal fructose absorption and, therefore, essential to absorb an important dietary component of modern diets. However, regulatory pathways that modulate GLUT5 expression are not well understood.

In response to fructose, GLUT5 traffics to the apical cell membrane by the Ras-related protein in brain 11a (Rab11a), followed by fructose absorption by enterocytes. In the cytoplasm, fructose is phosphorylated by KHK, a step required for the increased expression of fructolytic genes, including *GLUT5*^34,35^. Intracellular fructose or its metabolites in turn stimulate the transcription factor carbohydrate responsive element-binding protein (ChREBP, also known as MLX-interacting protein-like (MLXIPL)), which promotes the uptake and conversion of excess carbohydrate by regulating glycolytic, lipogenic and fructolytic genes, including Glut5^36^. Recently, it was shown that the majority of ingested fructose is metabolized by the intestine, whereas only excess spills over to the liver^37^. In line with this, mice with an intestinal- but not hepatic ChREBP deficiency display fructose intolerance^38^.

Here we show that the human *GLUT5* promoter is strongly and specifically induced by LXRα. LXRα is a member of the NR family of ligand-activated transcription factors and its natural ligands are oxysterols which are oxidized derivatives of cholesterol. The LXR subfamily consists of two closely related isoforms, LXRα and LXRβ (NR1H3 and NR1H2) that are highly conserved between humans and rodents. LXRα is restricted predominantly to tissues that are active in lipid metabolism, including liver, intestine and adipose tissue. In contrast, LXRβ is much more widely expressed and is found in almost every tissue examined. Physiologically, LXRs act as cholesterol sensors and promote the transcription of genes involved in cholesterol homeostasis including intestinal cholesterol absorption and reverse cholesterol transport. Besides their role in cholesterol homeostasis LXRs also regulate carbohydrate metabolism, for example through direct regulation of the bifunctional enzyme 6-phosphofructo-2-kinase/fructose-2,6-bisphosphatase (PFK2/FBP2), which has a role in hepatic glycolysis and gluconeogenesis^39^.

LXRs modulate gene transcription by heterodimerising with the Retinoid X Receptor (RXR) and binding to direct repeat of the consensus hexanucleotide sequence AGGTCA with a spacing of 4 (DR4, AGGTCAnnnnAGGTCA), also termed LXRE in the promoters of target genes. We observed a specific induction of the human *GLUT5* promoter by LXRα and this could be further stimulated by addition of ligand. In contrast, LXRβ was a much less potent activator of *GLUT5* and could also not be further stimulated by ligand. It is generally believed that the LXR isoforms have largely overlapping target genes, however, isoform-specific functions have also been found. Activation of genes involved in Srebp1c-mediated hepatic *de novo* lipogenesis is mostly contributed to LXRα activation while target genes in peripheral tissues are regulated by both isoforms^40^. When we further characterized the regulation of the human *GLUT5* promoter by LXR, we identified a DR4 type LXRE located at −429 to −374 bp upstream of the ATG start codon. Interestingly, this specific DR4 element in the human *GLUT5* promoter was previously described to be regulated by THR. This interaction was first described by Matosin-Matekalo *et al*., who located a functional DR4 site in the human *GLUT5* promoter sequence responsible for THR regulation^19^. Our data is consistent with their finding and we also show that LXRα and THR can competitively bind this DR4 site. An interaction between LXR and THR on a DR4 element in promoters of target genes has previously been described for other genes, explaining extensive crosstalk between these receptors at different levels^41–43^. In addition to competitive DNA binding, there may also be other interactions between LXR and THR such as competition for cofactors.

*Glut5* is also expressed in pre-adipocytes where it is involved in the adipogenesis, resulting in reduced adiposity in eWAT of *Glut5*^−/−^ mice and potentially contributing to the increased (visceral) adiposity caused by high-fructose consumption^44^. Under normal conditions, the physiological relevance of peripheral fructose metabolism is thought to be limited since fructose is normally readily taken up and metabolized by the intestine, resulting in low systemic fructose levels^45^. Moreover, the rate-limiting enzyme KHK-C is absent or expressed at very low levels in adipocytes, so fructose metabolism is dependent on the inefficient KHK-A splice variant in adipose tissue^46^. Nevertheless, deletion of KHK-A resulted in an exacerbated metabolic phenotype in mice receiving 30% fructose via their drinking water, highlighting the relevance of peripheral fructose metabolism by KHK-A under conditions of excessive fructose consumption^47^. This supports the hypothesis that it is metabolically beneficial to divert hepatic fructose metabolism by increasing fructose uptake in adipocytes.

In mice we demonstrated that treatment with the synthetic LXR ligand T09 significantly stimulates *Glut5* expression in the small intestine and eWAT after 7 and 14 days of treatment, respectively. Importantly, this transcriptional upregulation of *Glut5* was accompanied by an induction of GLUT5 protein levels both in duodenum and eWAT after T09 treatment. In line with a direct activation of the *Glut5* promoter by LXRα, genomic analysis using ChIP-seq identified two LXR binding sites in the mouse *Glut5* promoter (Fig. S4b).

The fact that *Glut5* in eWAT was only increased after 14 days of treatment could also point towards a mechanistic pathway downstream of LXR activation, such as via regulation of ChREBP, which itself is an LXR target^48^. ChREBβ, but not α, was slightly increased in adipose tissue after 14 days T09 treatment. Intestinal ChREBα/β expression, however, was unchanged during LXR activation (Fig. S6). Besides, this delayed expression in adipose tissue was also observed for *Srebp1c*, which is only a direct target of LXR but not of ChREBP. To establish the potential ChREBP-dependent effects in response to LXR activation additional studies are needed. Finally, delayed induction in adipose tissue might also be explained by for example delayed accumulation of the ligand in this tissue.

LXR agonists have been extensively investigated for their therapeutic potential in a variety of diseases including atherosclerosis, inflammatory diseases, diabetes and Alzheimer’s disease^49^. Several synthetic LXR ligands are being investigated in clinical trials^50–52^, however, their development has been challenged by adverse effects such as severe hepatic steatosis and hypertriglyceridemia^53,54^. To address this problem a new generation of highly potent and selective LXR modulators is being developed that separate the benefits of the LXR-agonists from their unwanted side effects. For example, LXRα-selective antagonists or inverse agonists might be efficacious in the treatment of NAFLD without affecting reverse cholesterol transport^55^. On the other hand LXRβ-selective agonists are potentially useful to improve cognition in Alzheimer’s disease while avoiding stimulation of hepatic lipogenesis by LXRα, the predominant isoform in the liver^56^. GLUT5 expression is increased in the intestine and in skeletal muscle of T2D patients but also in certain cancers that are highly dependent on fructose uptake and metabolism, providing an alternative route for energy delivery during insulin resistance or to match increased energy demand caused by uncontrolled proliferation, respectively^28,29^. As a ligand-activated transcription factor LXRα might provide novel pharmacologic strategies for the selective modulation of GLUT5 activity in the treatment of metabolic disease as well as cancer.

## Materials and Methods

### Animals

Male mice of >99% C57BL/6J genetic background (Charles River, USA) between 8–12 wks of age were used in this study. Animals were housed in a light- and temperature-controlled facility (lights on from 7 a.m. to 7 p.m., 21 °C) with free access to water and standard chow (RMH-B, AB Diets). Animal experiments were performed with the approval of the local Ethics Committee for Animal Experiments of the University of Groningen. All experiments were performed in accordance with relevant guidelines and regulations (including laboratory and biosafety regulations).

### Animal experiments

Mice were randomized into four groups including three treatment groups and one control group. Mice were treated with the synthetic LXR ligand T0901317 (T09; Cayman chemical, USA; 0.015%; ~30 mg/kg/day) added to their chow diet for either 1 day, 1 week or 2 weeks. The control group received non-supplemented chow diet. Mice were anesthetized with isoflurane and euthanized by cardiac puncture followed by cervical dislocation. Tissues were collected and frozen in liquid nitrogen.

### Cell culture

CV-1 (Monkey kidney fibroblast cell line) and Hek293 cells (human embryonic kidney cell line) were cultured at 37 °C and 5% CO_2_ in Dulbecco’s Modified Eagle (DMEM) GlutaMAX^TM^ medium (Life Technologies, USA) with high D-glucose (4.5 g/L), pyruvate and supplemented with 10% heat-inactivated fetal bovine serum (FBS) (Life Technologies, USA), and 1% penicillin and streptomycin (p/s) (Life Technologies, USA). Cells were grown till 80% confluence and split at least twice a week.

### Plasmids, cloning and site-directed mutagenesis

Full length cDNAs for the murine nuclear receptors LXRα, LXRβ, RXRα, THRα and THRβ were cloned into the pcDNA3.1-V5H6 vector (Invitrogen, USA). The human *GLUT5* promoter (−900 to +3 bp relative to the transcriptional start site (TSS) inserted in a reporter plasmid (pGL4-luc)) was obtained from SwitchGear Genomics (USA). Human *GLUT5* promoter truncations 1–3 (−783, −429 and −374 to +3 bp relative to TSS, respectively) were generated by digestion of the human *GLUT5* promoter using, respectively, restriction enzymes *Bgl*I; *Acc65*I and *Bsa*I; *Pvu*II, *Hind*III and *Acc65*I (Promega, USA) followed by subcloning into pGL4-luc or by ligation using Rapid DNA ligation kit (Roche, Germany). Site-directed point mutations were introduced into the human *GLUT5* promoter by 2-step overlap-extension PCR using Expand High Fidelity PCR system (Roche, Germany) as described in literature^57^. Primers were designed to contain mutated nucleotides and restriction sites for *Sac*I and *Hind*III using the human *GLUT5* promoter construct as a template.

The mouse *Glut5* promoter (−1014 to +3 bp) was generated by PCR using C57BL/6 mouse genomic DNA as a template followed by cloning into the pGL4-basic luciferase vector using the restriction sites *Nhe*I and *Hind*III (Promega, USA). All constructs were validated by Sanger sequencing (BaseClear, Leiden).

### High-throughput NR promoter reporter screen

Promoters were tested for regulation by individual ligand-activated NR-members that heterodimerize with RXR (Table S1). For high-throughput screening, plasmids of NR/RXR heterodimers (15 ng NR + 15 ng RXR), promoter-reporters (30 ng) and internal controls (5 ng) pRL TK-Renilla (Promega, WI, USA) or pCMV-lacZ (Clontech CA, USA) were aliquoted in a volume of 10 μl/well into 384-well plates (Corning, 3707) using a 96-head liquid handling robot (Packard Evolution). The empty pcDNA3.1/V5-HisA vector (Invitrogen) was used to equalize the total amount of DNA transfected in all conditions. For reverse transfection, FuGENE HD (0.195 µl/well) and OptiMEM (4.805 µl/well) were mixed and 5 µl was added per well. After adding, the plate was gently shaken at RT and 35 µl of CV-1 cells (resuspended in phenol red-free DMEM medium with 10% charcoal-stripped serum) was added per well using a Titertek Multidrop 384 automated HT cell dispenser, making up a total volume of 50 µl per well. Plates were covered with a breathable seal and shaken gently before returning them to the incubator. The next day, 5 µl of ligand (Table S1) or control medium was added per well and incubated for another 24 h. The third day, plates were analyzed for luciferase activity using a luminometer (Wallac 1420 VICTOR2™ V, Perkin Elmer).

### Reporter assays

CV-1 cells were seeded in 48 wells plates and the medium was changed to DMEM GlutaMAX^TM^ medium (Life Technologies, CA, USA) with high D-glucose (4.5 g/L), pyruvate and supplemented with 10% heat-inactivated charcoal-stripped FBS (Life Technologies, USA), and 1% antibiotics (p/s; Life Technologies, CA, USA) before transfection. CV1 cells were transiently transfected with plasmids containing promoter reporters (100 ng) and NR cDNAs (50 ng unless otherwise stated) in OptiMEM (Life Technologies, USA) using FuGENE^®^ HD reagent (Promega, USA). The empty pcDNA3.1/V5-HisA vector (Invitrogen) was used to equalize the total amount of DNA transfected in all conditions. Renilla (3 ng, pRL TK-Renilla, Promega, USA) and LacZ (10 ng, pCMV-LacZ, Clontech, USA) were co-transfected in all experiments to normalize for variation in transfection efficiency. Figures are shown with LacZ as internal control unless otherwise stated. NR ligands were added 24 hours after transfection, and luciferase activity was determined 48 hours after transfection using a Dual-Luciferase Assay kit (Promega, USA) and measured using the SynergyH4 hybrid reader (BioTek, USA). Ligands used were T0901317 (1 μM T09, Cayman Chemical in DMSO), triiodothyronine (1 μM T_3_ in absolute ethanol, Sigma-Aldrich, USA) and 9-cis-Retinoic acid (1 μM 9cRA in DMSO, Sigma-Aldrich, USA), unless otherwise stated in the figure legend. Ligands were tested with the empty vector and showed no effects on luciferase signal in absence of their NR. In addition, vehicle solvents were tested and had no influence on the luciferase signal in these reporter experiments. The human *GLUT5* promoter activity measured as luciferase signal is influenced by factors present in FBS and, therefore, stripped FBS is used in our luciferase reporter assays (Fig. S1). Results shown are representative for at least three independent experiments.

### Gene expression analysis

RNA was isolated from the liver, skeletal muscle, WAT and the proximal (duodenum) and middle (jejunum) part of the small intestine of the mice. Total RNA was isolated using Trizol (Life Technologies, USA) and reverse transcribed into cDNA using M-MLV reverse transcriptase kit (Invitrogen) and random nanomer primers (Sigma-Aldrich, USA). For quantitative PCR (qPCR), cDNA was amplified using Hi-ROX SensiMix™ SYBR green (Bioline, London, UK) and StepOnePlus™ Real-Time PCR System (Applied Biosystems, CA, USA). Intron-spanning primers were used to prevent the amplification of genomic DNA (primer sequence in Table S2). Ribosomal protein RPLP (*36B4*) was used as a reference gene to normalize the data according to the ∆∆Ct method.

### Protein analysis

For immunoblot analysis, whole-cell lysates were obtained using M-PER Mammalian Protein Extraction Reagent (Thermo Fisher Scientific, USA) supplemented with Complete inhibitor cocktail tablet (Roche, Germany). Protein concentrations were quantified using the RC DC protein assay (Bio-Rad, USA). Protein samples were subjected to SDS-PAGE (4–15% gradient gels, Biorad, USA) and transferred to nitrocellulose using Trans-Blot® TurboTM transfer system (Bio-Rad, USA). After blocking for 1 hour at room temperature (RT) in TBS containing 0.1% Tween and 2% milk powder, membranes were incubated overnight with primary antibodies at 4 °C. Primary antibodies used in this study are: anti-Glut5 (Millipore cat# 07-1406, 1:1000) and anti-GAPDH (Calbiochem, Darmstadt, 1:5000). Antibodies were detected by incubating the blot with horseradish peroxidase (HRP)-conjugated donkey anti-rabbit (Life science, NA934, 1:2500) or rabbit anti-mouse (Dako, p0260, 1:5000) IgG for 1 hour at RT. Protein bands were detected using ChemiDoc (Bio-Rad, USA) after incubation with the SuperSignal West Pico Chemiluminescent Substrate System (Thermo Fisher Scientific, USA). GLUT5 levels in samples were calculated relative to GAPDH using Image lab 3.0 software (Bio-Rad Laboratories, USA).

### Statistical analysis

GraphPad Prism 5.00 software package (GraphPad Software, USA) was used to perform statistical analysis. For reporter assays, a representative experiment is shown (n = 3) and significance was determined using a t-test when comparing two groups and a one-way ANOVA followed by a Tukey’s post-hoc test when comparing more groups. Significance in animal experiments was determined using the nonparametric Mann Whitney U-test when comparing two groups or the Kruskal-Wallis H-test when comparing more groups. The Dunns post-hoc test was performed after a significant Kruskal-Wallis test. All values are given as means ± SD unless stated otherwise. Significance is indicated as *P < 0.05, **P < 0.01, ***P < 0.001.

## Supplementary information

Supplementary data
