# Supplementary material for: Identification of the fructose transporter GLUT5 (SLC2A5) as a novel target of nuclear receptor LXR
Source: Sci Rep. 2019 Jun 26;9:9299. doi: 10.1038/s41598-019-45803-x (PMC6594926; doi:10.1038/s41598-019-45803-x)
Supplement: Supplementary file 1 — Supplementary data [file 41598_2019_45803_MOESM1_ESM.docx]

**Identification of the fructose transporter GLUT5 (*SLC2A5*)**

**as a novel target of nuclear receptor LXR**

Irene Zwarts^1^, Tim van Zutphen^1^, Janine K. Kruit^1^, Weilin Liu^1^, Maaike H. Oosterveer^1^, Henkjan J. Verkade^1^, N. Henriette Uhlenhaut^2,3^

and Johan W. Jonker^1^*^#^*

*^1^Section of Molecular Metabolism and Nutrition, Department of Pediatrics, University of Groningen, University Medical Center Groningen, Hanzeplein 1, 9713 GZ Groningen, The Netherlands. ^2^Molecular Endocrinology, Institute for Diabetes and Obesity, Helmholtz Zentrum München, Business Campus Garching, Parkring 13, Garching 85748, Germany.* *^3^Metabolic Biochemistry and Genetics Ludwig-Maximilians-Universität München Gene Center, Munich, Germany. ^#^To whom correspondence should be addressed. E-mail:* [*j.w.jonker@umcg.nl*](mailto:j.w.jonker@umcg.nl)*.*

**Supplementary data**

**Table S1.** **Nuclear Hormone Receptor Superfamily**

| **Class** | **Official**  **name** | **Common name** | **Short**  **name** | **Accession NR (mouse)** | **ligand** | **Ligand conc.** |
| --- | --- | --- | --- | --- | --- | --- |
| 1A | NR1A1 | Thyroid hormone receptor α1 | THRα1 | NM_178060 | T_3_ | 1 μM |
|  | NR1A1 | Thyroid hormone receptor α2 | THRα2 | NM_178060 | T_3_ | 1 μM |
|  | NR1A2 | Thyroid hormone receptor β1 | THRβ1 | NM_009380 | T_3_ | 1 μM |
|  | NR1A2 | Thyroid hormone receptor β2 | THRβ2 | NM_009380 | T_3_ | 1 μM |
| 1B | NR1B1 | Retinoic acid receptor α | RARα | NM_009024 | TTNBP | 100 nM |
|  | NR1B2 | Retinoic acid receptor β | RARβ | NM_011243 | TTNBP | 100 nM |
|  | NR1B3 | Retinoic acid receptor γ | RARγ | NM_011244 | TTNBP | 100 nM |
| 1C | NR1C1 | Peroxisome proliferator activated receptor α | PPARα | NM_011144 | WY14643 | 30 μM |
|  | NR1C2 | Peroxisome proliferator activated receptor δ | PPARδ | NM_011145 | GW501516 | 100 nM |
|  | NR1C3 | Peroxisome proliferator activated receptor γ | PPARγ | NM_011146 | BRL49653 | 1 μM |
| 1H | NR1H2 | Liver X Receptor β | LXRβ | NM_009473 | T0901317 | 1 μM |
|  | NR1H3 | Liver X Receptor α | LXRα | NM_013839 | T0901317 | 1 μM |
|  | NR1H4 | Farnesoid X Receptor | FXR | NM_009108 | GW4064 | 1 μM |
|  | *NR1H5* | *mouse FXRβ* | *FXRβ* | NM_198658 | *-* |  |
| 1I | NR1I1 | Vitamin D Receptor | VDR | NM_009504 | calcipotriol | 100 nM |
|  | NR1I2 | Pregnane X Receptor | PXR | NM_010936 | PCN | 1 μM |
|  | NR1I3 | Constitutive Androstane Receptor | CAR | NM_009803 | TCPOBOP | 250 nM |

**Table S2.** Primer sequences used for cloning and qPCR analysis.

| **Primer name** | **Sequences 5’ - 3’** |
| --- | --- |
| hGLUT5 promoter Mutation Fw | ctgtTCCAATCAGATTCAGTGgtgtcatgtagggtggagg |
| hGLUT5 promoter Mutation Rv | caccACTGAATCTGATTGGAacagattgggccactagagacc |
| mGLUT5 promoter Fw/NheI | catgctagcCATTGCTACCCGAGCGGATCTAC |
| mGLUT5 promoter Rv/HindIII | cgtaagcttCATCGTTGCTCTGCAAGTGCAGCC |
| mRPLP0 Fw | CTG TTG GCC AAT AAG GTG CC |
| mRPLP0 Rv | GGA GGT CTT CTC GGG TCC TA |
| mGLUT5 Fw | CGA AAA ACC TAC GAG GGG CT |
| mGLUT5 Rv | CTG GCC AGC CAT CCT CAT TT |
| mSrebp1c Fw | GGA GCC ATG GAT TGC ACA TT |
| mSrebp1c Rv | CCT GTC TCA CCC CCA GCA TA |
| hRPLP0 Fw | CGT CCT CGT GGA AGT GAC AT |
| hRPLP0 Rv | TAG TTG GAC TTC CAG GTC GC |
| hGLUT5 Fw | GGC TTC TCC ATC TGC CTC ATA G |
| hGLUT5 Rv | GAT GAC ACA GAC GAT GCT GAT GT |
| hSrebp1c Fw | GGA TTG CAC TTT CGA AGA CAT G |
| hSrebp1c Rv | AGC ATA GGG TGG GTC AAA TAG G |

**Fig. S1. Transcriptional regulation of the human *GLUT5* promoter by nuclear receptors RXR, LXR and THR.** Luciferase reporter assays with a human *GLUT5*-Luc plasmid co-transfected with **a)** RXRα (40 ng DNA) and LXRα (40 ng DNA), with and without LXR ligand T09, with DMEM containing heat-inactivated FBS or charcoal-stripped heat-inactivated FBS in CV1 cells (y-axis; fold induction to the basal transcription activity of the human *GLUT5* promoter measured and corrected with the internal standard LacZ).

**Fig. S2.** Luciferase reporter assays using **a)** human *GLUT5*-Luc plasmid and **b)** mouse *GLUT5*-Luc plasmid which were co-transfected with RXRα and LXRα including a LXR ligand T09 in Hek293AD cells (y-axis; fold induction to the basal transcription activity of the *GLUT5* promoter and corrected with the internal standard LacZ).

**Fig. S3.** Competitive reporter assay with a human *GLUT5*-Luc plasmid co-transfected with **a)** RXRα, LXRα and increasing amount of THRβ (0, 50, 100 ng) with LXR ligand T09 **b)** RXRα, THRβ and increasing amount of LXRα (0, 50, 100 ng) with THR ligand T_3_ in CV1 cells (y-axis; fold induction to the basal transcription activity of the human *GLUT5* promoter and corrected with the internal standard LacZ).

**
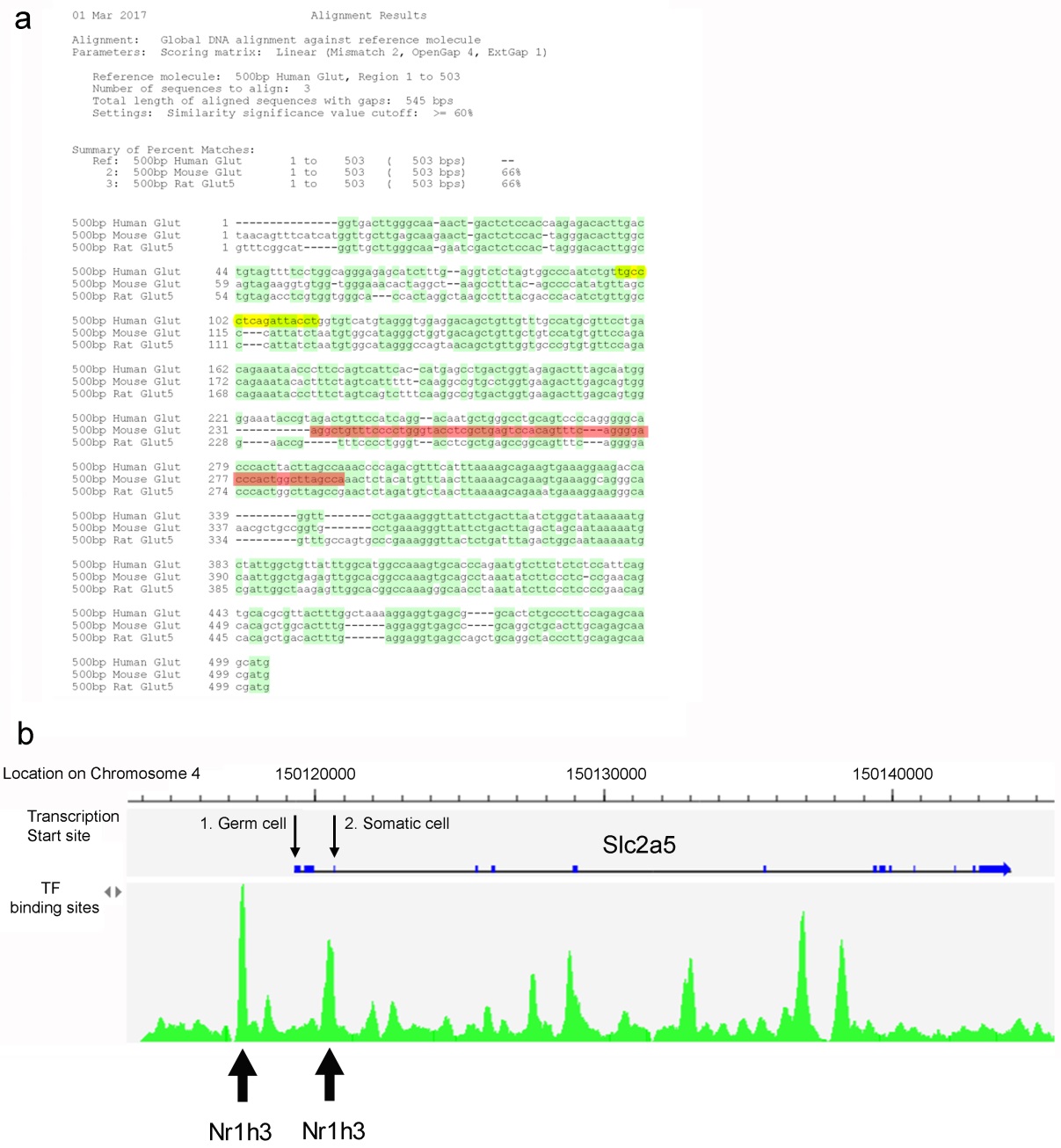
**

**Fig. S4. Genomic analysis of LXR binding to the GLUT5 promoter. a)** Alignment of the human, mouse and rat *GLUT5* proximal (-500/+3 bp) promoter. Highlighted in yellow is the LXR/THR-responsive DR4 element and in red the LXR binding sequences (indicated by arrows in panel b) identified by LXR ChIP-seq. **b)** ChIP-seq analysis of primary mouse macrophages region, showing the transcription *Glut5* start sites (TSS) for (1) germ- and (2) somatic cells and reported transcription factor (TF) binding sites in green, including LXRα (Nr1h3) at the indicated positions (bold arrows). The binding site located 155 bp upstream of the TSS in somatic cells is indicated in red in the alignment in a.

**
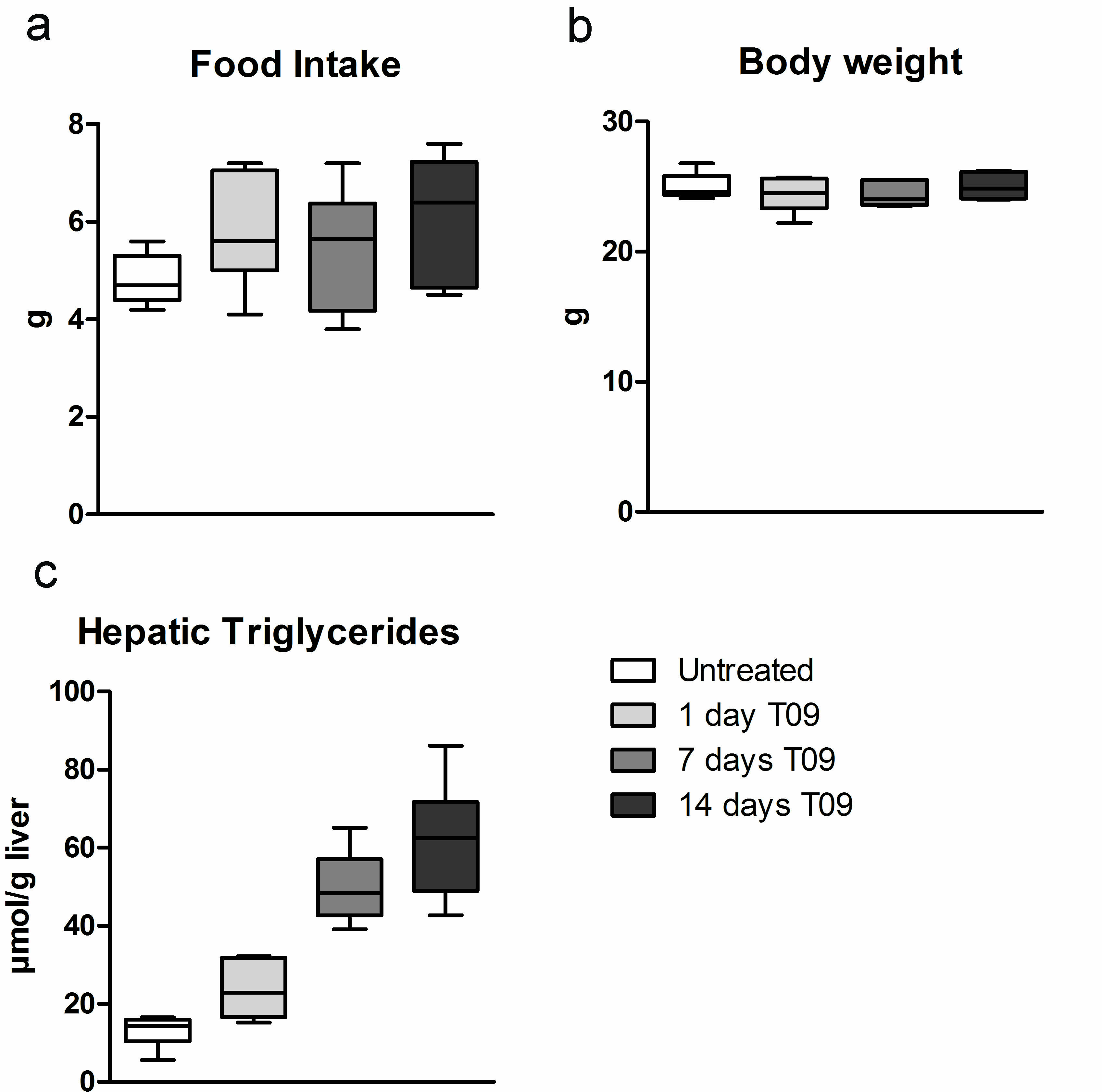
**

**Fig. S5** Effect of LXR-activation on **a)** food intake, **b)** body weight and **c)** hepatic triglycerides in wild-type mice treated for 1, 7 or 14 days with 0.015% T0901317 supplemented in their standard chow diet (n = 5-8). Data are presented as boxplots (middle line, median; box, 25th-75th percentiles; whiskers, 5th-95th percentiles).

**Fig. S6.** Effect of LXR-activation by T0901317 treatment on ChREBPα and ChREBPβ expression in mice. mRNA levels of ChREBPα and ChREBPβ in a,b) duodenum and c,d) eWAT in wild-type mice treated for 1, 7 or 14 days with 0.015% T09 supplemented in their standard chow diet (n = 5-8, y-axis; normalized to 36B4 universal reference gene). Data are presented as boxplots (middle line, median; box, 25th-75th percentiles; whiskers, 5th-95th percentiles).
